# Indoor Positioning System (IPS) Using Ultra-Wide Bandwidth (UWB)—For Industrial Internet of Things (IIoT)

**DOI:** 10.3390/s23125710

**Published:** 2023-06-19

**Authors:** Fuhu Che, Qasim Zeeshan Ahmed, Pavlos I. Lazaridis, Pradorn Sureephong, Temitope Alade

**Affiliations:** 1 Department of Computing and Engineering, University of Huddersfield, Huddersfield HD1 3DH, UK; fuhu.che@hud.ac.uk (F.C.); p.lazaridis@hud.ac.uk (P.I.L.); 2 College of Arts, Media and Technology, Chiang Mai University, Chiang Mai 50200, Thailand; pradorn.s@cmu.ac.th; 3 Department of Computer Science, School of Science and Technology, Nottingham Trent University, Nottingham NG11 8NS, UK; temitope.alade@ntu.ac.uk

**Keywords:** UWB, indoor positioning system, localization, machine learning, NLoS

## Abstract

The integration of the physical and digital world has become increasingly important, and location-based services have become the most sought-after application in the field of the Internet of Things (IoT). This paper delves into the current research on ultra-wideband (UWB) indoor positioning systems (IPS). It begins by examining the most common wireless communication-based technologies for IPSs followed by a detailed explanation of UWB. Then, it presents an overview of the unique characteristics of UWB technology and the challenges still faced by the IPS implementation. Finally, the paper evaluates the advantages and limitations of using machine learning algorithms for UWB IPS.

## 1. Introduction

With advancements in wireless communication technology, sixth generation (6G) is bringing advanced technologies, such as mm-Wave [1,2,3], unmanned aerial vehicles (UAVs) [4,5,6], tera-hertz (THz) communications [7,8,9], intelligent reflecting surfaces (IRS) [10,11,12], non-orthogonal multiple access (NOMA) [13,14,15], etc., to overcome the limitations of prior wireless generations. Indoor positioning systems (IPSs) and location-based services have become a fundamental requirement for many Industrial Internet of Things (IIoT) applications [16,17,18]. For global positioning in the outdoor environment aspect, global navigation satellite systems (GNSSs), such as GPS, GLONASS, and the BeiDou navigation satellite system, are widely used and can achieve positioning accuracy within a 4.9 m radius in clear, open spaces [19,20]. However, despite these systems bringing great convenience to human life, the positioning accuracy decreases significantly in indoor environments or dense urban areas, where satellite signals are heavily attenuated when they pass through building walls, leading to multipath conditions or complete signal blocking [21,22,23,24].

IPSs require high precision, often with centimeter-level accuracy, and are becoming increasingly important for various applications, such as personal navigation in airports and shopping malls, warehouse management and security, machine, and asset tracking in smart factories, health monitoring in hospitals, personal information delivery tracking, and commercial wheeled-robot control in industry areas [17,25,26,27]. However, indoor environments are still challenging due to the heterogeneous nature and the presence of various obstacles that cause variations in signal and noise levels, making high-precision localization a difficult task. To address this challenge, various technologies have been employed, including RFID, BLE, Wi-Fi and ZigBee [25,26,28,29]. These commercially available technologies can provide IPSs with an accuracy of the meter order, which may suffice for some applications. However, UWB technology is emerging as a more promising technique for high-accuracy indoor localization, capable of achieving centimeter-level accuracy in larger-coverage areas in harsh environments with only a few reference anchors and an effective radiated power (ERP) of 2W [30,31,32]. UWB has several attractive properties, including high channel capacity because of its extremely wide bandwidth, which enables low transmission power, and the extremely short time duration of the pulses (typically nano- or picoseconds), which reduces multipath fading. The robustness to multipath effects and high temporal resolution also make UWB a suitable technology for high-precision localization by enabling precise ranging based on time of arrival (ToA), time difference of arrival (TDoA) and two-way time of arrival (TW-ToA) techniques [33,34].

Several survey articles have been written on indoor localization, focusing on different technologies and their strengths and weaknesses. Zafari et al. [35] conducted a comprehensive survey of different types of indoor positioning techniques, considering factors, including the efficiency, hardware cost, reception, latency, scalability, and localization accuracy. They also highlight the challenges that need to be addressed to achieve accurate positioning systems. Sattarian et al. [36] reviewed the use of data mining technology in an indoor positioning system for IoT applications and how data mining can help overcome the challenges. Alarifi et al. [37] conducted a detailed analysis, including the strengths, weaknesses, opportunities, and threats, of UWB positioning technologies. Additionally, Hayward et al. [38] explored the potential applications of IPSs in the industrial sector. However, these articles do not provide a detailed discussion on the effects of the non-line-of-sight (NLoS) signal and some existing cutting-edge machine learning (ML) algorithms that can be used to classify NLoS signals. There is a need for a comprehensive review that summarizes these wireless technologies for IPSs and ML algorithms for UWB to provide a better understanding of future research directions.

Firstly, this work presents an up-to-date applications of IPSs and the wireless technologies that can be used for IPS. Then, UWB characteristics and principles of position estimation methods and algorithms are discussed. The second objective of this article is to present a review of the NLoS signal’s effects on the UWB positioning system. Finally, the article discusses the existing ML algorithms used to classify or mitigate the positioning error caused by NLoS signals and the main challenges for further work. The key contributions of this work are as follows:

1. This work provides a detailed survey of the most common wireless communication-based technologies for IPSs and evaluates these technologies using an evaluation framework to highlight their pros and cons.
2. This paper provides a detailed discussion of various principles of position estimation methods that can be used for IPS, and highlights the advantages and limitations of using algorithms for UWB IPSs.
3. In addition, this paper presents a detailed explanation of UWB. Then, it presents an overview of the unique characteristics of UWB technology and the challenges still faced by IPS implementation.
4. This paper also presents an exhaustive review of the non-line-of sight (NLoS) signal’s effects on the UWB positioning system and discusses the existing ML algorithms used to classify or mitigate the positioning error caused by NLoS signals and the main challenges for further work.
5. Finally, this work surveys and discusses the emerging state-of-the-art ML-based research efforts in solving the challenge associated with NLoS effects for the UWB presented and summarizes the existing popular ML algorithms for UWB IPS NLoS classification and mitigation, such as k-NN, SVM, DT, NB, and NN.

This paper is organized as follows: Section 2 describes the techniques used for localization in IIoT. Section 3 describes the characteristics of UWB. Section 4 explains UWB IPSs and the principles of localization. Section 5 provides the definition of the NLoS signal and importance of applying ML algorithms for the UWB system and presents the existing ML algorithms and their comparisons. A complete section on future research directions, challenges, and limitations is discussed in Section 6. Finally, the summary and conclusions are presented in Section 7.

## 2. Localization in IIoTs

In this section, the progress and the key benefits of IPSs are discussed. Popular wireless technologies employed for IPSs for IIoT, such as Wi-Fi, BLE, ZigBee, RFID, and UWB, are revisited followed by analysis of the evaluation metrics, such as accuracy, coverage, power consumption, etc., for such technologies.

### 2.1. Indoor Positioning System, IPS

**Figure 1:**
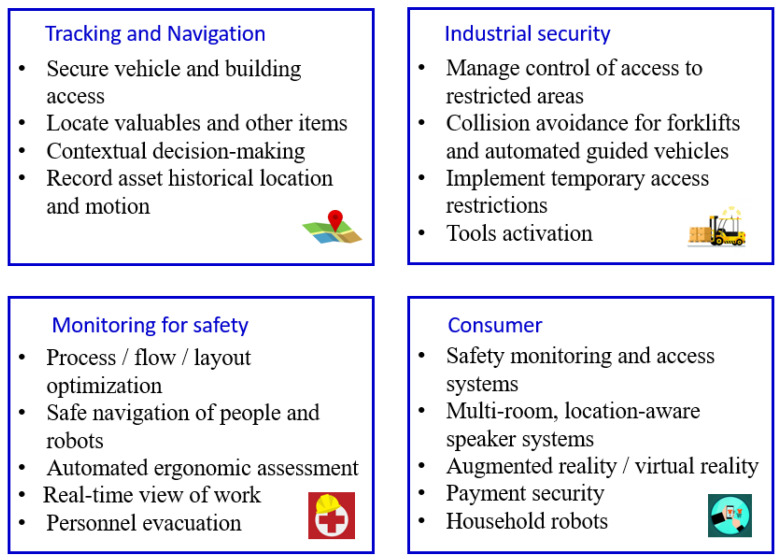
Key benefits of indoor positioning system (IPS).

IPSs have enabled various navigation applications that highly require the instant location of a person and any objects in real time, uniformly localizing the mobile device or objects in an indoor environment [39,40]. It has opened up various new possibilities in industrial, consumer IoT markets and healthcare. In industrial environments, there is a growing interest in IPSs for logistics and manufacturing to ensure the precise navigation of automatic robots, tracking personal tools and equipment in large warehouses and factories. In such environments, device movement can be potentially dangerous, so automatic device tracking has become a fundamental aspect of Industry 4.0 for safety [41,42]. Additionally, there is an increasing legal requirement for the continuous tracking of coal miners in underground mines due to the increasing number of disasters with many fatalities. These positioning systems can help to connect front-end workers and improve safety [43]. In the consumer IoT market, real-time and reliable location information can digitalize and optimize virtually every aspect of asset and data management for efficiency and security in manufacturing operations and working spaces [44]. Context-aware location-based marketing is undergoing a great change and shows potential improvements in both sales and profits side in e-commerce [45]. This type of marketing strategy enhances the shopping experience in real time by considering the buyer’s social profile, shopping history, feedback requirements, spending pattern, history of navigation, online behavior, and so on. Finally, IPSs are attracting attention in healthcare due to the need to improve service quality, such as monitoring biomedical equipment location or guiding patients in crowded hospitals [46]. For example, in the case of emergency patients, doctors can track the safety and mobility of patients instantly. Other applications, such as map construction and route planning, are well suited for IPSs [47]. The map construction is a fingerprinting-based localization approach that consists of an offline stage and an online stage. Fingerprints are in the form of radio frequency (RF) signal strength, which is collected and stored in the offline stage. The location pair consists of exhaustive records of the serviced area. For the online stage, location estimation is estimated by comparing the collected testing records to the stored training fingerprints. Furthermore, route planning for intelligent localization and navigation systems is another extremely useful technology with both military and commercial applications. For standard route planning algorithms, it generates a minimum cost route based on a predetermined cost function. The beneficial opportunities for IPSs are summarized in Figure 1.

### 2.2. Communication Technologies for Indoor Positioning System

In this following subsection, the most common existing wireless communication technologies that are widely applied for indoor localization system [48,49,50], such as RFID, Wi-Fi, Bluetooth, ZigBee, and UWB, are briefly presented and discussed.

**Figure 2:**
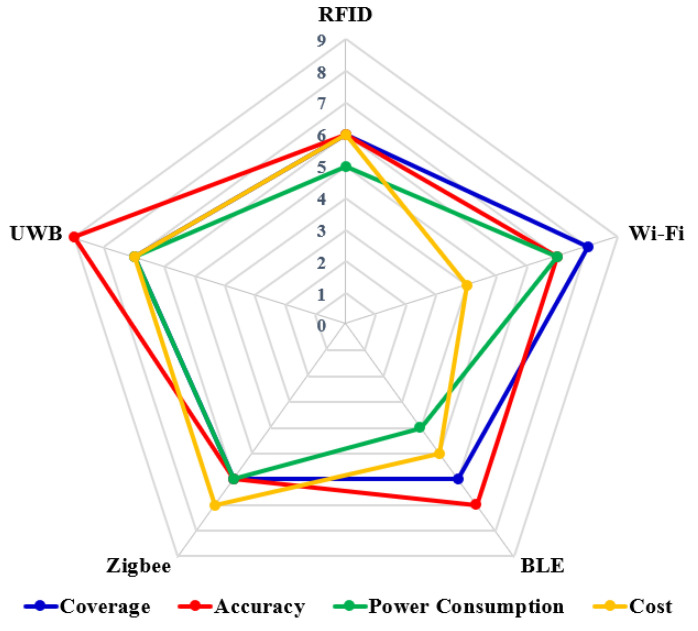
Wireless technologies for IPS comparison in terms of coverage, precision, energy efficiency and cost.

- **Wireless Fidelity, (Wi-Fi):** Wi-Fi, a widely used wireless networking technology, operates on the IEEE 802.11 standard and uses radio frequency bands of 2.5 GHz for IEEE 802.11b and 5 GHz for IEEE 802.11a [49]. Many smart devices, such as smartphones, tablets, and audio players, are Wi-Fi enabled, making Wi-Fi-based IPSs more practical and cost effective. In a typical large indoor area, such as office buildings, universities, and malls, the widespread distribution of Wi-Fi hotspots provides a complete building coverage. Wi-Fi-based localization systems are typically based on fingerprinting the radio signal strength indicator (RSSI) and have an accuracy range of 1–10 m [50]. Wi-Fi offers a reception range of about 100 m, and its low infrastructure cost makes it a practical option for IPS. Its reasonable accuracy, availability, large coverage, high data rate, and widespread support in many devices make Wi-Fi a suitable choice for IPSs.
- **Bluetooth Low Energy, (BLE):** Bluetooth communication technology operates in the radio frequency range from 2.402 GHz to 2.480 GHz [51]. It is designed for short-range communication between devices and has become a competitive technology in IPSs due to its characteristics of cost effectiveness, very low power consumption, long battery life, high security, and communication efficiency [48,52]. Bluetooth-based localization solutions typically use the RSSI-based range-estimate technique. The latest version of Bluetooth, known as BLE, has a data rate of 24 Mbps, and the signal range coverage can reach 70–100 m with the high power efficiency, making it ideal for use in public-space areas, such as airports or shopping centers [53].
- **ZigBee:** ZigBee is a wireless communication protocol based on the IEEE 802.15.4 standard that is designed for personal-area networks that are cost effective, have low data rate, and are energy efficient. It operates on different frequency bands, including 868 MHz in Europe, 915 MHz in the USA, and 2.4 GHz in other regions. It can be easily applied for IPSs with a coverage range of up to 100 m [54], which is ideal for most indoor environments, including buildings and underground structures. The energy-efficient feature of ZigBee makes it a suitable choice for IPSs in terms of low power consumption.
- **Radio Frequency Identification, (RFID):** RFID is a key technology enabling the real-time monitoring of objects. It involves data transfer and storage, and operates on backscattering communication, which consists of a RFID reader, RFID tags, and data processing system [55,56]. The RFID reader emits frequency pulses that are received by the RFID tags, and the data are processed with the help of a chip embedded in the tags. The RFID tag contains three different types, which are active, passive and semi-active. Active RFID tags, which have an internal battery, are used in various applications and operate in ultra-high frequency ranges with a coverage range of up to 100 m [57,58,59]. RSSI information between the RFID tags and the reader is used to estimate the range and localization, but this information is easily affected by multi-path, noise, and changing channel conditions in indoor environments. Factors such as node density, antenna type, and frequency used can also impact the accuracy of the system. As a result, active RFID technology may not provide sub-meter-level precise accuracy of the positioning system, but it is still popular due to its low cost, ease of implementation, miniaturize size, and low power consumption [60,61].
- **Ultra-WideBand, (UWB):** UWB technology has gained popularity in precision indoor positioning systems due to its advantages over narrowband-based technologies, such as Bluetooth and Wi-Fi. Some factors of UWB include a very large bandwidth, very high data rate, short signal transmission length, low transmission energy, and high penetration capability [62,63,64,65]. These characteristics are also very important for high precision indoor localization accuracy. Currently, UWB technology has already received significantly attention in industry, as many companies have started to adopt it for precise tracking and navigation systems. For example, the iPhone-13 from Apple contains UWB for precise location tracking, and the Samsung Galaxy Note 20 Ultra uses UWB as a digital key for doors and cars. The structure of a UWB signal is based on the IEEE 802.15.4a–2011 standard, which involves the signal transmission of extremely short pulses within a very large bandwidth, specifically from 3.1 to 10.6 GHz [65,66,67], rather than broadcasting on separate frequencies. Due to its large bandwidth and short pulses, UWB systems are highly precise and secure, and are less susceptible to multipath interference and fading.
- **Evaluation Metrics of different technologies:** Evaluation metrics can explain the parameters which affect the performance of a technology. The metrics of different wireless indoor positioning technologies are summarized in Figure 2. The technologies are compared in terms of accuracy, energy efficiency, range coverage, and cost. The maximum metric achievable by a technology is 9. From the figure, it can be observed that UWB is highly accurate as compared to BLE, Wi-Fi, RFID and ZigBEE. However, the lowest power consumed is by BLE followed by RFID, ZigBEE, UWB, and Wi-Fi. Finally, it can be concluded that there is a trade-off when selecting an appropriate technology, and depending upon the application, the most suitable technology should be chosen.

## 3. UWB Characteristics

### 3.1. UWB Definition

Developed in the 1970s, UWB is a wireless radio technology developed initially for classified applications by the US military, but the focus has shifted to impulse radio UWB (IR-UWB) with ongoing research, according to [64]. To distinguish UWB from narrow-band signals, the Federal Communications Commission (FCC) has defined that UWB can be treated as a radio frequency (RF) signal: firstly, when the occupying bandwidth is greater than or equal to 500 MHz, and secondly, the fractional bandwidth of UWB has to be greater than 20% of the center frequency [65] and expressed as

(1) $$B_{f}=2\times \left(\frac{f_{H}-f_{L}}{f_{H}+f_{L}}\right),$$

where $B_{f}$ is a dimensionless frequency-independent indicator, and $f_{H}$ and $f_{L}$ mean the higher and lower cut-off frequencies at −10 dB of the UWB pulse spectrum. $B_{f}$ and band ratio $B_{r}$ help determine the types of communication. Table 1 summarizes the communication band usage scenario by classifying the communication system as a narrow-band, wide-band or ultra-wideband system.

### 3.2. Pulse Shape

The impulse radio version of UWB technology is commonly known as a pulse-based UWB system. In this system, the UWB pulse, often referred to as a Gaussian doublet, utilizes a square pulse due to its ease of generation through the simple on/off switching of a transistor. However, as previously noted, UWB pulses are typically measured in nanoseconds or picoseconds, and the rapid on/off switching makes the pulse shape non-rectangular but rather helps approximate it as a Gaussian function [17,27]. The basic modulation methods can be applied for UWB to encode information including pulse position modulation (PPM), burst position modulation (BPM), pulse amplitude modulation (PAM), and on–off keying (OOK) [68,69]. Generally, higher-order modulations can achieve higher throughput or good spectral efficiencies by enabling more bits to be sent per symbol [68,69,70]. PPM is a common method for creating an *M*-ary system and is easy to implement but requires very good time resolution to modulate the pulses. In BPPM, the pulses can be sent at the same rate, and the changes of UWB shape depend on the transmitted value [71]. PAM modulation is based on using the pulse amplitude to encode information and allows for the use of an arbitrary number of different pulses. The OOK method can be also considered a particular case of PAM, whereas only a binary set of pulses is allowed. A UWB signal can be represented as a UWB signal transmitted by the help of *K* pulses; the pulses are within a period of $T_{p}$ that consists of certain frames, where each information symbol is considered a UWB signal [62,63].

### 3.3. Advantages of UWB

**Figure 3:**
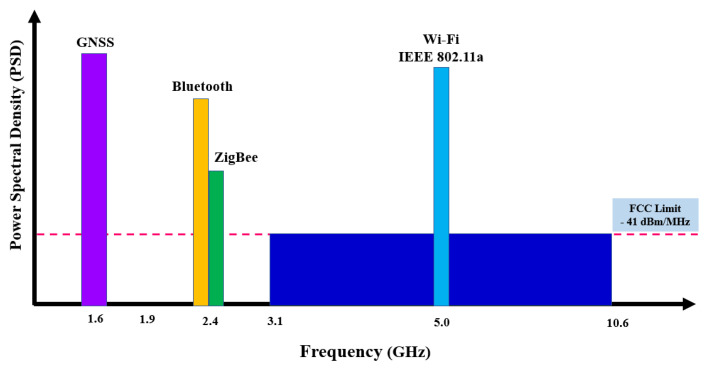
Comparison of the attributes of UWB spectrum with various positioning technologies.

- **Large Channel Capacity:** According to Hartley–Shannon’s capacity formula, the channel capacity increases linearly with bandwidth [66]. In such a case, the availability of some bandwidth which operates in typical gigahertz for UWB signals suggests that data rates of gigabits per second (Gbps) can be achieved. UWB technology transmits very short pulses within an extremely large bandwidth from 3.1 to 10.6 GHz, which provides a significant bandwidth advantage and a short duty cycle. As a result, UWB offers a larger capacity and higher data rates, making it an excellent choice [68,69,71].
- **Simple transceiver architecture and low cost:** UWB uses carrierless waves to transmit data [68,69,71,72]. As a result, carrier oscillators are not required in order to transmit the carrier frequency for the signal transmission. This eliminates the requirements for a carrier recovery stage for the receiver side, and the UWB transceiver does not require modulators, demodulators, or intermediate frequency components [68,69,71,72]. This simplicity in the UWB transceiver architecture makes it more lightweight and beneficial compared to narrowband signals. Furthermore, the system power consumption is significantly reduced due to these characteristics. Additionally, the low complexity of the UWB system and the smaller chip sizes reduce the cost of the system.
- **Multipath Immunity and Low Power Spectral Density (PSD):** Multipath refers to the phenomenon in which an electromagnetic signal travels through various paths during transmission due to factors such as signal reflection, signal absorption, diffraction, and scattering of energy by the presence of objects in the environment [65,66,67]. UWB communication systems have a large bandwidth, which allows them to operate at high data rates, making them highly robust. They are also capable of performing well in the condition of low signal-to-noise ratio (SNR) communication channels, providing immunity against multipath conditions. This factor makes UWB communication ideal for indoor positioning applications under NLoS conditions. Furthermore, UWB systems have good anti-multipath performance and are not sensitive to channel attenuation. The signal transmitting of UWB is of a low average power spectral density because of the short-pulse nature of the transmission, which places it within the noise floor (typical −40 dBm/MHz), thus allowing for less transmitter power consumption, increased power efficiency, and resistance against jamming and interception as shown in Figure 3.

### 3.4. IEEE 802.15.4 UWB Physical Layer (PHY)

**Figure 4:**
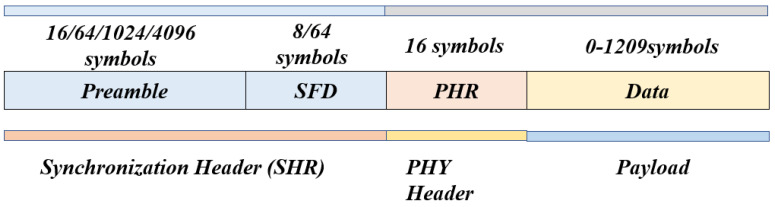
UWB PHY frame.

The UWB physical layer (PHY) waveform operates on a signaling scheme called the impulse radio, which uses band-limited data pulses. Three different frequency bands are defined by the IEEE 802.15.4 standard: the sub-gigahertz band, low band and high band. The sub-gigahertz band has one channel that spans from typically 249.6 MHz to 749.6 MHz. The low band covers the spectrum from typically 3.1 GHz to 4.8 GHz and consists of four channels (channel 1 to channel 4). The high band occupies the range 5.8 GHz to 10.6 GHz and includes 11 channels (channel 5 to channel 15), which are listed in Table 2. These optional channels can increase the communication range and positioning accuracy, as well as improving resistance to multipath interference. Figure 4 shows the UWB packet format. The packet or frame consists of three parts: a synchronization header (SHR), which includes the preamble sequence and the start of frame delimiter (SFD), a physical layer header (PHR), and the data portion. The preamble is made up of pulses that are used to detect the frame and are composed of two parts: the preamble and the SFD. The SFD marks the end of the preamble and the start of the PHY header, and it is used to determine the accurate frame reception timestamp that is essential for precise localization. The UWB PHY contains a mandatory short SFD of 8 symbols for the default and medium data rates, and an optional SFD of 64 symbols for the nominal low data rate of 110 kbps. The number of symbols in the preamble can be classified as 16, 64, 1024, or 4096 symbols, and the options are defined based on the application requirements. One of these preamble lengths has to be supported in order for the device to comply with the UWB standard.

## 4. UWB Indoor Positioning System

### 4.1. Architecture of UWB-Based IPS

**Figure 5:**
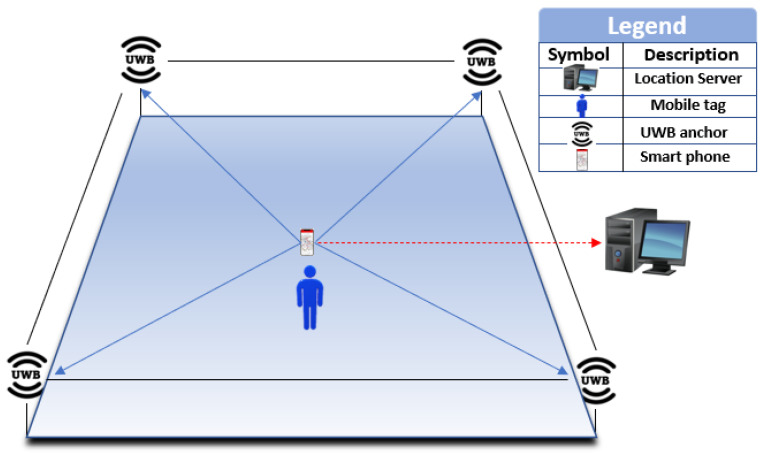
Basic elements of UWB positioning system.

Figure 5 depicts a typical UWB-based indoor localization system consisting of two types of nodes- anchors with known positions and tags with unknown positions. The system also includes a location server for sensor processing data and an interface device for viewing the positioning results. The localization process involves setting one of the anchors as the reference point and using the time-of-flight (ToF) technique to estimate the distance between each anchor and tag. Trilateration or multi-angulation techniques are then used to determine the coordinates of the tag in a 2D or 3D environment, depending on the number of anchors available. To improve accuracy in complex indoor environments, additional units, such as navigation frameworks, network gateways, user interfaces, multi-sensor technologies, and NLoS mitigation methods, are required. An NLoS detection algorithm is used to detect the presence of NLoS signals in the measurement data, and the model obtained from this algorithm is then used to refine the positioning algorithm. The choice of the NLoS detection algorithm and the positioning algorithm depends on the specific application requirements and the properties of the environment to achieve accurate positioning using the UWB IPS.

### 4.2. UWB Ranging Algorithms

**Figure 6:**
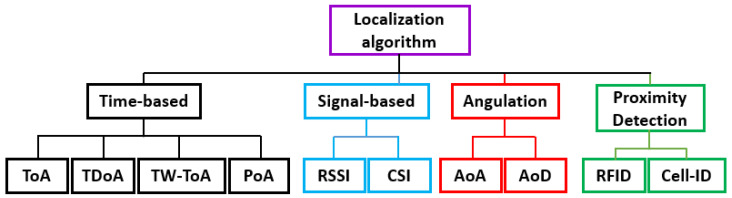
Classification of localization algorithms.

IPS ranging algorithms can be basically classified into four categories based on their underlying principle—time, signal, angulation, and proximity detection—as illustrated in Figure 6. Time-based algorithms include time of arrival (ToA), time difference of arrival (TDoA), two-way (TW)-ToA and phase of arrival (PoA). Signal-based algorithms rely on RSSI and channel state information (CSI). Angulation-based algorithms use angle of arrival (AoA) and angle of departure (AoD) to determine position. Proximity detection-based techniques use RSSI and Cell-ID. ToA, TW-ToA, TDoA, AoA, and RSSI are commonly used with UWB in the literature. In the upcoming sections, we will discuss these methods in detail.

**Figure 7:**
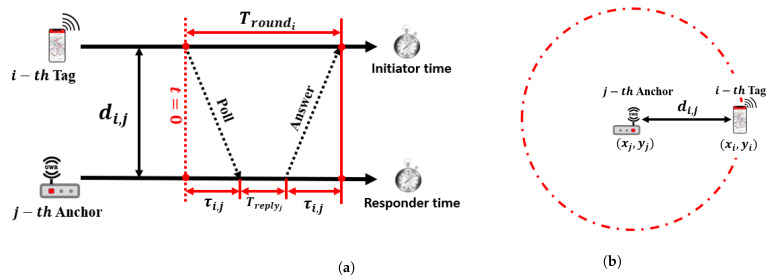
ToA positioning algorithm. (**a**) Signal propagation time calculations. (**b**) Estimated range scheme.

**Figure 8:**
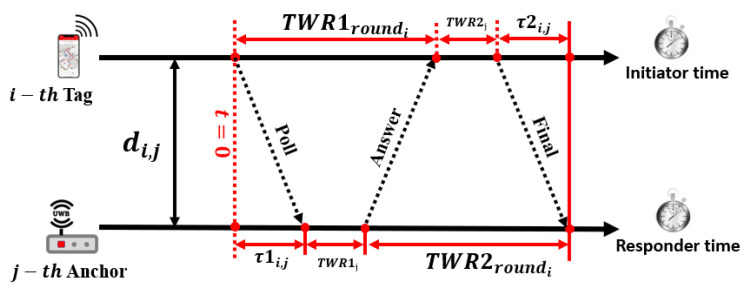
TW-ToA positioning algorithm.

**Figure 9:**
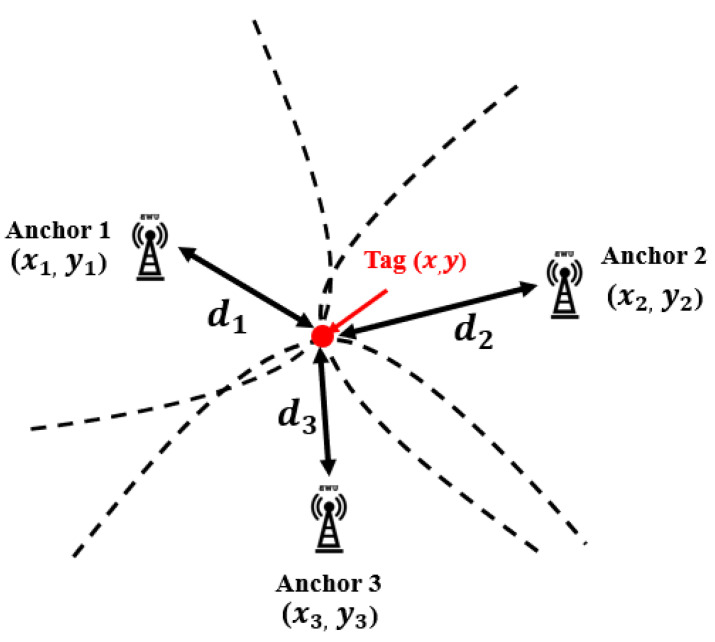
TDoA positioning algorithm.

**Figure 10:**
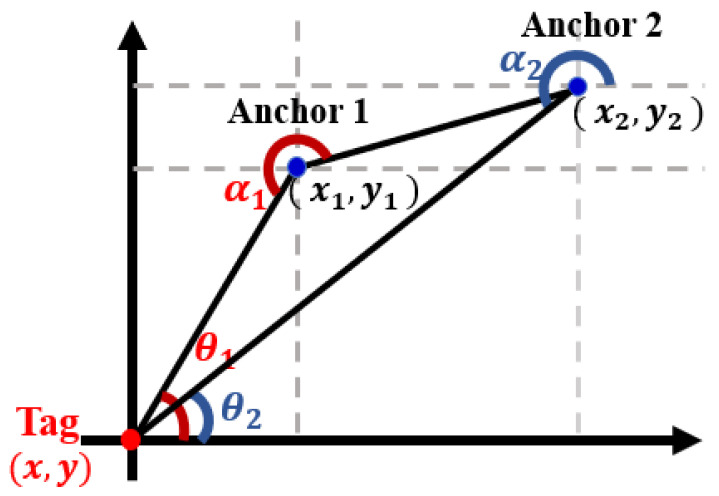
AoA positioning algorithm.

**Figure 11:**
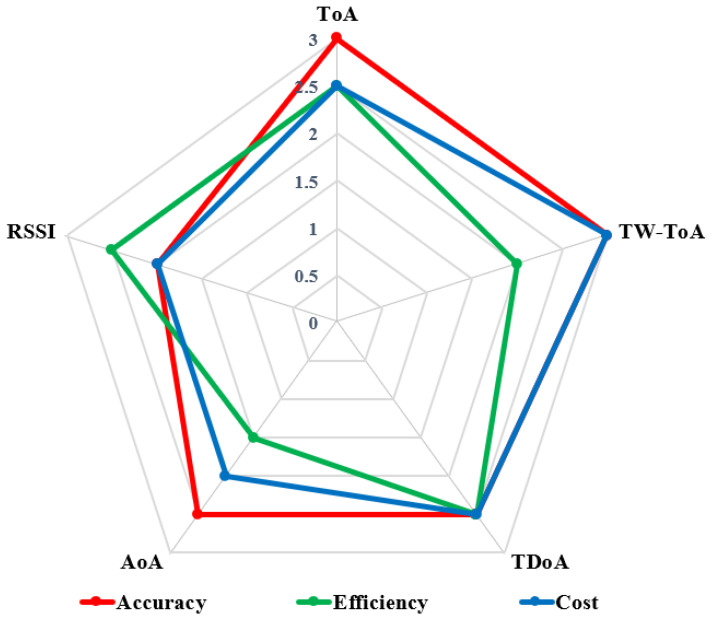
Comparison of the different positioning algorithms in terms of accuracy, cost and efficiency.

- **Time of Arrival (ToA):** According to [67], the majority of UWB-based IPSs employ the ToA algorithm to determine the position of mobile tags. This is because the positioning algorithm is simple to implement and provides high accuracy. The ToA algorithm measures the flight time between the anchors and tags and calculates the estimated range between each anchor and tag as illustrated in Figure 7a. The clocks of the anchors and tags are synchronized precisely, and a timestamp is sent from the *i*-th tag to the *j*-th anchor. The *j*-th anchor then sends back a reply after processing the timestamp, with $T_{reply_{j}}$ denoting the processing time of the *j*-th anchor. Let $T_{round_{i}}$ be the total time taken by the *i*-th tag, the total propagation time for $\tau_{ij}$ the *j*-th anchor, and the *i*-th tag can be expressed as
  (2) $$\tau_{ij}=\frac{T_{round_{i}}-T_{reply_{j}}}{2}.$$
  The estimate range $d_{i,j}$ between the *i*-th anchor and *j*-th tag can be determined as
  (3) $$d_{i,j}=c\times \tau_{i,j},$$
  where $c=3\times 10^{8}$ m/s representing the speed of light.
  The above equations reveal that the ToA algorithm is susceptible to errors resulting from time measurements. A 1μs time measurement error can result in an error of 300 m using RF wave velocity. Therefore, the ToA algorithm requires precisely synchronized clocks for both anchors and tags, which can be challenging in terms of hardware design and cost effectiveness. After determining the estimated range between each anchor and tag, trilateration theory can be employed to calculate the position of the mobile tag using the ranges obtained from more than three anchors at fixed known locations as shown in Figure 7b. To estimate the position of the *i*-th tag with respect to the *j*-th anchor, let us set the coordinates of the *j*-th anchor as $\left(x_{j},y_{j}\right)$ and being fixed in known positioning. Set the coordinates of the *i*-th tag as $\left({\hat{x}}_{i},\hat{y}i\right)$, where $\left(\hat{\cdot }\right)$ denotes the estimated position. The position of the tag is estimated by intersecting circles (in 2D) or spheres (in 3D) with radii d(i,j) and d(i,j,k), respectively. The optimal position of the tag $\left({\hat{x}}_{i},{\hat{y}}_{i},{\hat{z}}_{i}\right)$ can be obtained by applying the least-squares solution and the minimum mean square error estimation algorithm
  (4) $$\begin{array}{c} \left({\hat{x}}_{i},{\hat{y}}_{i},{\hat{z}}_{i}\right)=\min_{\left(x_{i},y_{i},z_{i}\right)}\sum_{j=1}^{4}{\left[d_{j}-\sqrt{{\left({\hat{x}}_{i}-x_{j}\right)}^{2}+{\left({\hat{y}}_{i}-y_{j}\right)}^{2}+{\left({\hat{z}}_{i}-z_{j}\right)}^{2}}\right]}^{2}. \end{array}$$
- **Two-Way Time of Arrival (TW-ToA):** The ToA method described above can offer high positioning accuracy but requires precise synchronization of the anchors and tags, which can be challenging to implement. Alternatively, the TW-ToA method shown in Figure 8 can be used to measure the signal propagation time τ and eliminate the synchronization requirement. The total propagation time for $\tau_{ij}$ between the *j*-th anchor and *i*-th tag can be calculated using the TW-ToA method and is expressed as
  (5) $$\begin{array}{ccc} \tau_{ij} & = & \frac{1}{4}\left(TWR1_{round_{i}}+TWR1_{j}+TWR2_{round_{i}}+TWR2_{j}\right), \end{array}$$
  where $TWR1_{round_{i}}$ and $TWR2_{round_{i}}$ are the two-way return time and $TWR1_{j}$ and $TWR2_{j}$ are the responding time of the anchor and the tag, respectively, as shown in Figure 8.
- **Time Difference of Arrival (TDoA):** TDoA is another time-based measurement algorithm related to ToA and TWR-ToA. The principle of this algorithm is to measure the difference in arrival time between two signals as shown in Figure 9. While the anchor still requires precisely synchronized clocks, the tags do not need to be as precisely synchronized compared to the ToA method. This leads to high-power efficiency, as only one transmission message is required from the tag to the anchor. The location of the mobile tag can be obtained from the intersection of multiple hyperbolas. Consider that the anchors are located at $(x_{i},y_{i}),i=1,2,3.$ and the coordinates of the tag are $\left(\hat{x},\hat{y}\right)$. The distance between the target and the reference base station can be expressed as a difference in arrival time, given as
  (6) $$\sqrt{{\left(\hat{x}-x_{i}\right)}^{2}+{\left(\hat{y}-y_{i}\right)}^{2}}=c\left(t_{i1}-t_{i2}\right),\;i=1,2,3.$$
- **Angle of Arrival (AoA):** The AoA algorithm, as shown in Figure 10, estimates the position of a mobile object based on angle measurements obtained by antenna arrays at the receiver side. The phase difference between two anchors is used to calculate each angle measurement, and the location of the mobile object can be determined from the intersection of the angle lines. In a two-dimensional Cartesian coordinate system, two anchors are located at $(x_{i},y_{i}),i=1,2$, and the coordinates of the mobile object are $\left(\hat{x},\hat{y}\right)$. The angles related to anchor-i from the standpoint of the mobile object are $\theta_{i},i=1,2.$ The angles measured by the anchors are denoted as $\alpha_{i},i=1,2.$ The location of the tag can be formulated as
  (7) $$\begin{array}{c} y_{i}-\hat{y}=\left(x_{i}-\hat{x}\right)\tan\left(\theta_{i}\right),\;i=1,2. \end{array}$$
  where $\theta_{i}$ and $\alpha_{i}$ have the following relation:
  (8) $$\begin{array}{c} \theta_{i}=180^{\circ }-\alpha_{i},\;i=1,2. \end{array}$$
  The target’s location $\left(\hat{x},\hat{y}\right)$ can be figured out by solving the equation.
- **Received Signal Strength Identification (RSSI):** To further expand on the RSSI algorithm, location fingerprinting involves collecting a database of RSSI values at known locations in the environment, known as “fingerprints”. When a mobile tag enters the environment, its RSSI values are compared to the fingerprints in the database to determine its location. This approach can improve the accuracy of the RSSI-based positioning system, but it requires significant effort to build and maintain the fingerprint database. Additionally, changes in the environment, such as moving objects or changes in building materials, can impact the accuracy of the system. Overall, RSSI-based algorithms can provide a low-cost solution for indoor localization, but their accuracy can be impacted by various environmental factors. In addition, these algorithms may not be suitable for applications that require high precision, such as industrial automation or autonomous vehicle navigation. The theoretical relationship between received signal strength and distance is as follows:
  (9) $$d_{i}=d_{0}{\left(10^{\left(\frac{P\left(d_{i}\right)-P_{t}-PL\left(d_{0}\right)+x_{\sigma }}{10}\right)}\right)}^{\frac{-1}{n}}$$
  where $d_{0}$ is the reference distance, $P_{t}$ and $PL(d_{0})$ are transmitted power and pass loss at the reference point, and $x_{\sigma }$ is a Gaussian random variable with zero mean that represents shadow fading and the path loss exponent.
- **Comparison of Positioning Algorithms:** Table 3 summarizes the advantages and disadvantages of the mentioned positioning algorithms. These positioning algorithms are compared in terms of accuracy, efficiency and cost in Figure 11. From the figure, it can be observed that TDoA, TWR-ToA, and ToA have the highest accuracy. RSSI followed by ToA and AoA have the lowest implementation cost, while ToA followed by TDoA and TWR-ToA have the highest efficiency. From the figure, it can be concluded that there is a trade-off when selecting the positioning algorithms, and depending upon the requirements, the positioning algorithm should be selected and preferred.
  Finally, to conclude this section, the current advances for UWB positioning algorithms in the literature are summarized in Table 4. The table categorizes each paper concerning the publication year, positioning algorithm applied, and the basic description explaining the rationale and methodology for each paper.

## 5. Detection in UWB Positioning Algorithms

**Figure 12:**
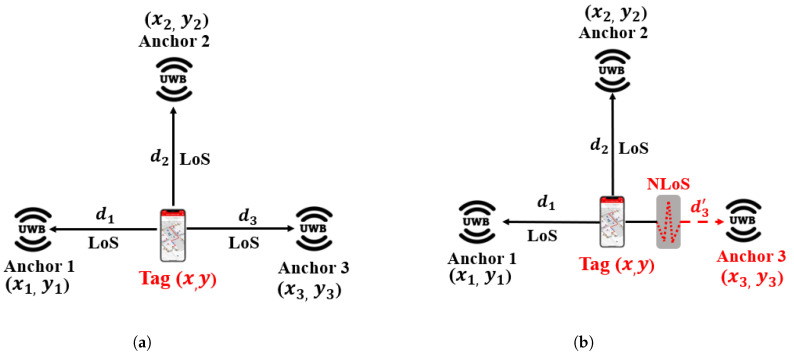

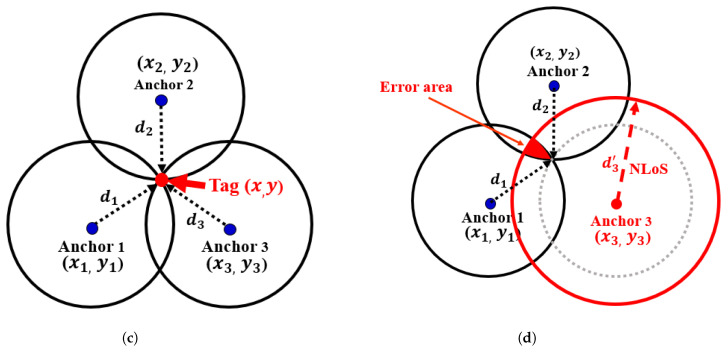
Detection of UWB positioning system. (**a**) Signal propagation in LoS scenario. (**b**) Signal propagation in NLoS scenario. (**c**) Positioning algorithm in LoS scenario. (**d**) Positioning algorithm in NLoS scenario.

Generally, in a UWB IPS, the signals are classified as either a LoS or NLoS signal [27,78,79,80,81]. There are some papers where the signals are classified as having quasiLoS (QLoS); see [82,83] and references therein. The detection of the UWB positioning system is classified as LoS and NLoS in Figure 12. In LoS conditions, where there is a clear environment and there are no obstacles between the anchor and the tag, the estimated range between each anchor and tag ($d_{1},d_{2},d_{3}$) can be calculated accurately, allowing the trilateration theory to be applied and the position of the tag to be accurately obtained as shown in Figure 12c. However, in NLoS conditions, where the signal is attenuated or refracted by obstacles causing a positive bias, the distance measurement for anchor 3 ($d_{3}^{{}^{\prime }}$) is estimated inaccurately. This causes the circles to overlap as shown in Figure 12d, resulting in the tag’s location being in any highlighted area rather than an accurate location point. As a result, the localization accuracy of the tag is seriously affected. Therefore, from Figure 12, it can be observed that the trilateration positioning algorithm suffers from positive bias NLoS errors. This positioning error can be solved by employing a joint approach of employing empirical and ML models. ML models can be used to classify these LoS and NLoS conditions to improve the accuracy of the positioning algorithm. Let us now look into ML models in detail for NLoS classification.

### 5.1. Machine Learning for UWB In NLoS

**Figure 13:**
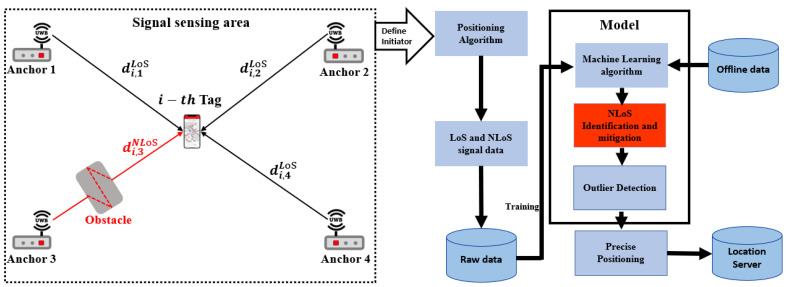
Blocks of machine learning NLoS detection for UWB precise positioning process.

To improve the accuracy of UWB positioning systems, specific NLoS mitigation techniques are required for various applications. Figure 13 illustrates a block diagram of a complete UWB precise IPS, which starts by fixing the anchors for the coordinate system and locates the mobile UWB tags within the indoor environment. The collected raw data will be used by an additional processing for NLoS detection before sending to the positioning system. The additional processing for NLoS detection is performed using an ML classification algorithm that has been pre-trained with the raw measurement data. This model is used to mitigate the NLoS effects. The UWB IPSs require a coordinate system with a reference point ref=(0,0,0) to provide relative 3D positions for both anchors $a_{q}$ and tags $t_{r}$:

(10) $$a_{q}\in \left\{a_{1},a_{2},\cdots ,a_{Q}\right\},$$

(11) $$t_{r}\in \left\{t_{1},t_{2},\cdots ,t_{R}\right\},$$

where *Q* is the total number of anchors (fixed node with known coordinates) and *R* is the total number of tags (mobile node with unknown coordinates) in the UWB IPS. The position of an anchor and a tag within the UWB localization system can be represented as

(12) $$a_{q_{pos}}=\left(a_{q_{x}},a_{q_{y}},a_{q_{z}}\right),$$

(13) $$t_{r_{pos}}=\left(t_{r_{x}},t_{r_{y}},t_{r_{z}}\right),$$

where *x*, *y*, and *z* are the Cartesian coordinates relative to the reference point, respectively. To calculate $t_{r_{pos}}$, the *r*-th tag $t_{r}$ will measure its range with each anchor $a_{q}$. The ground truth range $\Delta a_{q}t_{r}$ between $a_{q}$ and $t_{r}$ can be calculated using the Euclidean distance formula given as

(14) $$\Delta a_{q}t_{r}=\sqrt{{\left(a_{q_{x}}-t_{r_{x}}\right)}^{2}+{\left(a_{q_{y}}-t_{r_{y}}\right)}^{2}+{\left(a_{q_{z}}-t_{r_{z}}\right)}^{2}}.$$

As the target is to estimate the position of $t_{r}$, the x,y,z coordinates are not available to the UWB systems, while the x,y,z coordinates of each anchor $a_{q}$ are known. In UWB systems, $\Delta a_{q}t_{r}$ is determined by measuring the ToF between anchor $a_{q}$ and tag $t_{r}$. Specifically, in this research, we focus on ToA using the arrival time of three packets. Two packets are sent from tag $t_{r}$ to $a_{q}$ and one vice versa, which eliminates any processing time differences due to the influence of crystal inaccuracies, timing offsets, or processing delays. The result is a time of arrival which can be used to calculate $\Delta a_{q}t_{r}$ in LoS conditions as follows:

(15) $$ToA_{a_{q}t_{r}}=\frac{\Delta a_{q}t_{r}}{c},$$

where *c* is the speed of light (m/s).

In NLoS conditions, the ToA is longer due to the absence of a direct path and presence of multipath signals in the environment. Before calculating this error, the received UWB signal needs to be determined. The features, logged by the UWB receiver, represent information about the first- and multipath signal propagation between $a_{q}$ and $t_{r}$ and can be represented as follows:

(16) $$CIR_{a_{q}t_{r}}\left(t\right)=\sum_{s=1}^{S}\alpha_{s}\delta \left(t-\tau_{s}\right)+n\left(t\right),$$

where *t* indicates the timestamp for each value within the CIR. δ is the Dirac delta function, *S* is the number of multipath components, $\alpha_{s}$ and $\tau_{s}$ are the amplitude and the time delay of the *s*-th multipath components, respectively, and *n* represents the additive white Gaussian noise, respectively.

In the LoS condition, the first path (FP) component in the CIR corresponds to $ToA_{a_{q}t_{r}}$. However, in NLoS conditions, the detected first path by the UWB receiver is attenuated. As a result, in NLoS conditions, the calculated ${\hat{ToA}}_{a_{q}t_{r}}$ is inaccurate and is given as

(17) $${\hat{ToA}}_{a_{q}t_{r}}=CIR_{a_{q}t_{r}}\left(fp+s\right)=ToF_{a_{q}t_{r}}+\tau_{s},$$

where *s* is the first multipath above the noise floor, and earlier multipaths are not detected. The distance error (in meters) between $a_{q}$ and $t_{r}$ thus becomes

(18) $$e_{a_{q}t_{r}}=\tau_{s}\times c,$$

and the calculated range between $a_{q}$ and $t_{r}$ is given as

(19) $$\hat{\Delta }a_{q}t_{r}=\Delta a_{q}t_{r}+e_{a_{q}t_{r}}.$$

The goal of the UWB error correction ML model is to predict $e_{a_{q}t_{r}}$ accurately. To collect a dataset for the training process, the ground truth of $\Delta a_{q}t_{r}$ is required, together with the UWB estimated ranges $\hat{\Delta }a_{q}t_{r}$. Alternatively, ML models can also be used to predict whether the received $CIR_{a_{q}t_{r}}$ is LoS or NLoS and avoid $\hat{\Delta }a_{q}t_{r}$ in the UWB IPS. The ground truth of NLoS is typically based on a topology of the environment or based on visual indicators during the data measurement campaign.

### 5.2. Recent Advances in ML for NLoS Effects

In this section, we will present an overview of several research papers in the current literature related to UWB IPSs. The existing research work based on ML for UWB IPSs can be classified into two main categories: NLoS detection and NLoS error correction approaches. The primary goal of NLoS detection is to accurately classify the NLoS signal and then mitigate its effects. On the other hand, the primary objective of error correction is to identify the errors in the UWB ranges using precise ground truth, which can have a positive impact on the localization accuracy. Specifically, when there are at least four anchors available for 3D localization, an anchor selection algorithm can be used to mitigate range errors based on their NLoS detection before providing the ranges to the localization algorithm.

- **NLoS Classification:** In the UWB feature-based methods category, two papers are mentioned. Sang et al. [79] use three ML approaches to classify NLoS into multiple classes (LoS and NLoS) based on 12 extracted features, achieving an accuracy of up to 91.9% in the best case. Similarly, Zeng et al. [80] use a genetic algorithm to find the best combination of 18 features in an office environment, achieving an NLoS classification accuracy of 96%.
  In contrast, the non-feature-based methods category includes three papers. Jiang et al. [81] use a CNN to identify NLoS signals after denoising raw CIR data using a reversible transformation method, achieving an average accuracy increase of 27.9% for NLoS classification accuracy. Fan et al. [84] propose an unsupervised ML approach based on Gaussian mixture models to identify NLoS links from unlabeled data. Jiang et al. [85] use a CNN to extract non-temporal features from UWB raw CIR data, and then feed the features into long short-term memory for NLoS classification, achieving an accuracy of 82.14%.
  Compared to the feature-based methods, the papers based on raw CIR measurements provide superior performance for NLoS detection. However, the authors did not evaluate the performance of the proposed approaches in unseen environments, which limits their suitability in practical settings. In contrast, Park et al. [86] propose transfer learning based on neural networks (NN) and convolutional neural networks (CNN) to identify UWB NLoS signals in unseen environments.
- **NLoS Error Correction:** Besides NLoS detection, UWB error correction is mentioned in [87,88,89,90]. Similar to the NLoS approaches, some research papers focused on extracting features from the CIR data. The authors of [87] extracted the features based on distance measurement and received signal strength. Then the authors proposed local spatial feature extraction, temporal feature extraction, and position prediction to improve the positioning accuracy. Authors in [89] mainly focus on the UWB measured range associated with NLoS. A large dataset comprising of the measured distance and 7 different signal features are trained by an (ANN) to perform error prediction. The focus of [88] is on UWB feature-based error correction. Two classes of non-parametric regression techniques include a support vector machine and the Gaussian process and are applied by the authors to directly mitigate the ranging error in the physical layer, based on 6 signal features from the received waveform and the estimated distance. The fraction of residual errors less than 1m is increased from 63% to around 90% by using support vector machine- and Gaussian process-based mitigation. Finally, in paper [90], a semi-supervised autoencoder-based ML approach is proposed by the authors, based on raw CIR data, to achieve high IPS accuracy for low-cost edge devices. The results achieve 29% higher localization accuracy than state-of-the-art deep neural networks in complex environments.

### 5.3. Ml-Algorithms for UWB IPS

As mentioned, various types of ML algorithms have been proposed and used in a large range of applications for improving the IPS, especially for NLoS detection and error correction. Among the existing successive development of ML algorithms mentioned above, SVM, DT, NB, and NN have gradually improved the positioning accuracy and significant usefulness of IPSs. These algorithms are discussed in detail in this section.

**Figure 14:**
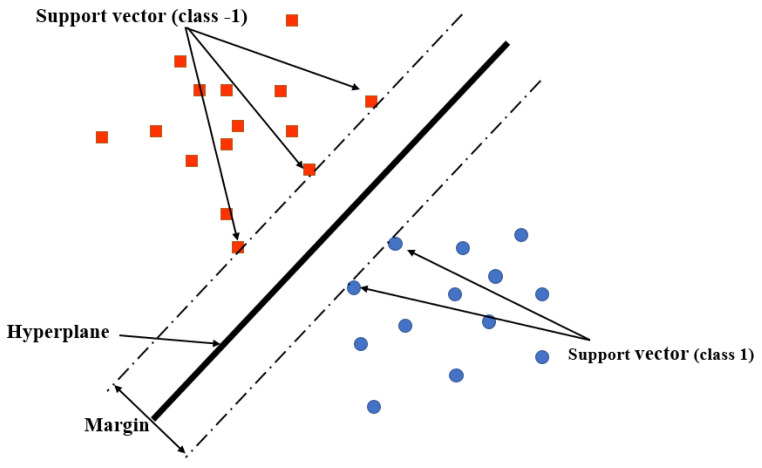
SVM algorithm.

- **k-Nearest Neighbors (k-NN):** k-NN is a type of the non-parametric-based supervised learning classifier that can be applied to both regression and classification. It typically uses the assumption of the data feature similarity that the data points can be found near one to another. The new data can be assigned a value based on how similarly the data match the points trained in the training set [91,92]. The advantages of k-NN algorithm can be summarized: Firstly, it is easy to implement and achieve high-accuracy results. Secondly, it is suitable for multi-label classification cation issues. In contrast, the disadvantage is that the algorithm requires large calculations, which can increase the memory overhead. Moreover, it provides relatively low-accuracy results when the sample is imbalanced [93,94,95].
- **Support Vector Machine (SVM):** SVM is a typical classic supervised ML algorithm that adopts the structural risk minimization principle to solve both classification and regression problems under high-dimensional space substitution [96]. It provides robust and superior performance without tuning several parameters due to it being based on the framework of statistical learning theory compared with other ML algorithms [97]. The main principle of the algorithm consists in estimating a hyperplane that can maximize the distance between the values of interest in each class. As shown in Figure 14, for a linearly separable dataset, there is only one separating hyper-plane with the largest geometric interval. Let us consider that a training dataset contains n points of the form $T=\left(x_{1},y_{1}\right),...,\left(x_{n},y_{n}\right)$, where the $y_{i}$ is labeled as 1 or −1. $x_{i}$ is the p-dimensional real feature vector and $x_{i}\in R^{n}$. The hyper-plane is the maximum margin determined to divide the group of points $x_{i}$ into group $y_{i}=1$ and group $y_{i}=-1$. The hyper-plane can be described by the following linear equation:
  (20) $$w^{T}x+b=0$$
  The advantages of SVM are as follows: firstly, strict mathematical theory support and strong interpretability due to the algorithm not requiring typical statistical methods, thus simplifying the usual classification and regression problems; secondly, it is easy to find key samples (i.e., support vectors) that are critical to the algorithm which can handle nonlinear classification and regression tasks; and thirdly, the calculation complexity of the algorithm depends on the number of support vectors instead of the dimensionality of the sample space, which can simplify the calculation process. In contrast, the disadvantage is that the training time is long due to the prediction running time being proportional to the number of support vectors.
- **Decision Tree (DT):** The DT algorithm is very suitable for large datasets with complex different features due to its ability to mimic human-like thinking for interpreting the data [98,99]. The advantage of DT is that it can break down the dataset into smaller subsets to operate the classification, which can minimize the classification error. In addition, DT can decide which attribute is the best at each tree node to ensure the high accuracy of the classification. The main advantage of decision tree learning is that it can minimize the error at the tree root due to it creating a single outcome by creating the tree at every leaf. Meanwhile, each tree root will also take a longer running time, which is the main disadvantage; therefore, it is not suitable for the application, which requires a fast response.
- **Naive Bayes(NB):** The Naïve Bayesian approach is based on the Bayesian principle for conditional probabilities [100,101]. The algorithm calculates the probability of each attribute value, then gives the values of each instance’s attributes. All instance probabilities are from the training set, and then the maximum probability is used to predict the class of the new instance. Given a new dataset of the form $<a_{1},a_{2},\cdots ,a_{n}>$, the predicted class for this instance dataset $l_{predicted}$ is
  (21) $$l_{predicted}=\underset{l\in L}{\arg\max}P\left(l\right)\prod_{i=1}^{n}P\left(a_{i}|l\right).$$
  where *L* is a vector of all attribute values, P(l) is the prior probability of *l*, P(a|l) is the probability of *l* given condition *a*, and P(l) is the prior probability of *a*.
- **Neural Network (NN):** In recent times, the neural network (NN), one type of deep learning (DL), has become relatively competitive for classification, clustering, pattern recognition and regression in various different areas [102]. It is an information management model that works in a similar way to the biological nervous systems function of the human brain [103]. The advantage of NN application is that it provides more accurate results due to complex natural systems with large numbers of inputs; thus, the network can generate the best possible result without the requirement of redesigning the output criteria [104]. In order to accomplish high-precision positioning, different NN models were proposed and evaluated for the implementation, such as the multi-layer perceptron (MLP) [105], radial basis function (RBF) [106] and generalized regression neural network (GRNN).

### 5.4. Performance of ML Algorithms

As stated, signal features can be extracted and used for NLoS classification and mitigation. This sub-section presents the results obtained by applying ML-based algorithms, such as KNN-, SVM-, DT-, NB-, and NN-based UWB signal features. For this experiment, 1000 LoS and 100 NLoS UWB signals are used [17,78]. The performance is compared with the running time, confusion matrix, and the correct rate (CR) for LoS and NLoS components. The findings are summarized in Table 5. The confusion matrix depicts metrics, true positive (TP), false positive (FP), false negative (FN), and true negative (TN), respectively. The best classification performance is achieved by the NN algorithm. TP =983 refers to LoS, resulting in a correct rate of 98.3%, which means 17 samples out of 1000 samples were inaccurately classified. The average running time of NN is 0.0606 s, which is better than that of the other considered algorithms. The precision and recall can reach 98.9% and 98.3%, respectively, and the overall accuracy is 97.5%. Compared to NN, NB follows closely and has similar performance, while the rest of the algorithms reach a slightly lower running time, and their classification accuracy is also lower in the considered experiment.

**Figure 15:**
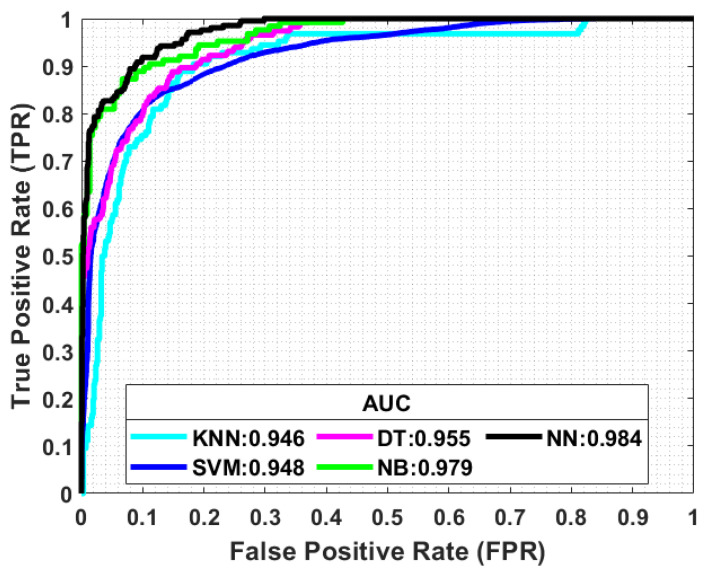
Receiver operating characteristics (ROC) and area under the curve (AUC) comparison of the state-of-the-art ML algorithms.

Figure 15 compares the receiver operating characteristics (ROC) curve and the area under the curve (AUC) of the discussed algorithms. The figure is plotted for the false positive rate (FPR) versus the true positive rate (TPR). The closer the curve to the upper left corner, the better the performance of the classifier. It is easily noticeable that the NN algorithm has the largest AUC of 0.984. Hence, it is superior to other algorithms and can classify the NLoS signals with the highest accuracy and, thus, improves the overall performance of the IPS.

## 6. Future Work, Challenges, and Limitations

Various ML-based algorithms have been proposed to mitigate the NLoS effects on IPS. However, the adaptation of ML-based methods for UWB indoor localization is still in its infancy, and some issues still need further investigation. The future directions can be summarized as follows.

### 6.1. Availability of Training Data

Both supervised and unsupervised ML algorithms are data dependent, requiring adequate data for training robust models. The amount and quality of the collected training data significantly affect the performance of ML algorithms. Achieving high accuracy localization becomes challenging when the training data are imbalanced, particularly when there are only a few NLoS as compared to LoS components in the data samples and vice versa. In situations where there is an imbalance in the dataset, existing ML algorithms face difficulties in training a robust classifier to classify the NLoS signal. To solve this problem, it is important to develop standard ML methods for training and predicting data that are independent, such as GD, GGD, and WNB algorithms [17,78,79].

### 6.2. Time Efficiency

The training time and response time of the ML model are also indispensable factors that influence IPS performance [107]. The training time means the time used for the ML algorithm to train the algorithm with an offline dataset and build the model; in the meantime, the response time means the time used for the model to predict the output for the given new testing data. In particular, for fast-moving objects, this could pose a challenge for the proposed methods due to the ML requiring a specific duration to process the NLoS signal. Additionally, the NLoS classification process may also require more processing time in dynamic scenarios. Therefore, it is crucial to test the proposed methods in such conditions to evaluate their performance and identify potential limitations. Future work needs to consider conducting experiments in dynamic scenarios to assess the effectiveness of the ML algorithms. The authors in [108] focused on proposing a dynamic video coding approach that utilizes dynamic video recording resolution adjustment on wearable cameras and Lyapunov-based video preprocessing on smartphones. The results show that the approach achieves up to 50% reduction in power consumption on smartphones and up to 60% reduction in average delay.

### 6.3. Extensibility and Scalability

There has been ongoing research addressing both NLoS detection and error correction to improve the performance of UWB IPS. However, it is worth noticing that the performance of the proposed approaches in the literature has not been evaluated in totally new environments yet. In such new environments, training datasets are collected using different techniques, and the collected data may vastly vary due to different factors, including device heterogeneity, such as different device topologies, the size of the room, the presence of objects, etc. Moreover, training separate models for each distinct environment requires considerable effort and time, and the UWB devices cannot remain in the same configuration [109]. Furthermore, the environments may change over time, necessitating frequent model updates. As a result, traditional ML algorithms are limited in their adaptability to entirely new environments. However, if collecting data and training different models for each unique environment, that would require considerable work and time (i.e., setting up the devices, performing large data collections, and executing the training model process). Even then, the environment may already change as time progresses, seriously requiring to update the models frequently [110]. Therefore, conventional techniques requiring new big datasets and completely new models are limited in their versatility to unseen and changing environments with different UWB configurations. To address this shortcoming, a transfer learning (TL) framework could be proposed, TL is an ML approach that can help with this task. Transferring the knowledge learned from one task to another similar task may not reliably transfer knowledge from a known domain to a new target domain with a satisfactory level of accuracy.

### 6.4. Variability

IPSs are hardware device based. The location estimation and NLoS mitigation are based on performance with user devices, which include limited storage capacity and computational capability. It is challenging to implement the ML model due to the models requiring computational and storage overhead for extracting complex signal features automatically from large amounts of collected data [111]. Moreover, a trained robust model could require retraining again when the definition, state and situation are changed in real-time localization systems. However, with the exponential growth in wireless networks, such as upcoming 6G and cloud facilities, it is expected for this computational burden to be handled successfully in the future and achieve numerous robust ML-based location services indoors.

### 6.5. Energy Consumption

Energy consumption has remained another concern for IoT-based positioning systems [8]. The trade-off between energy consumption and accuracy performance is a formidable challenge. For the high-accuracy positioning system, it is usually required to have more signal features for ML algorithms to reach higher NLoS classification accuracy with very high energy consumption of the systems, which could significantly reduce the battery life of IoT-based smart devices. Therefore, the technique and algorithm have to maintain a balance between positioning accuracy and energy requirements, especially crucial in mobile objects with fast movement. In such cases, ML algorithm optimization could be the approach, where the IoT-based positioning system continuously consumes a small amount of energy with remaining high-precision accuracy. The authors in [112] proposed an offline method to achieve minimum power consumption and an online solution to save energy for energy-aware video streaming on smartphones. Experimental results show that that the method can save energy, while achieving a better trade-off by implementing the online solution on Android-based smartphones.

### 6.6. Map Construction and Route Planning

The map construction technique collects the data of mapping from the physical space to the fingerprint space. It then trains the model and employs inverse mapping to estimate the location of the user or device. A unique challenge of map construction is that the measurements taken by training are not guaranteed to perform in the same physical space, and the measurements may also be obtained from different devices, resulting in an error [113]. On the other hand, route planning can play a critical role in determining the effective path to reach the end goal by considering various factors, such as distance, traffic, safety hazards, energy and time constraints, leading to an optimized journey with minimal cost to the device [114,115].

## 7. Summary and Conclusions

This paper delves into the current UWB IPS research. It starts with a detailed description of different wireless common technologies for IPSs, such as Wi-Fi, BLE, ZigBee, RFID and UWB, along with the research efforts in this regard. Then, it is followed by an evaluation of the advantages and disadvantages of localization algorithms for IPS. The paper also thoroughly surveys the unique characteristics of UWB technology and the challenges still faced by the IPS implementation. The state-of-the-art ML-based research efforts in solving the challenge associated with NLoS effects for UWB are also surveyed and discussed. Furthermore, k-NN, SVM, DT, NB, and NN techniques for ML-based UWB IPSs for indoor localization are discussed in detail as well as some of the obtained results of the UWB IPSs system development so that the ranging error can be reduced to less than 10 m. Finally, the paper identifies limitations and potential open problems for further research related to the successful deployment of ML-based localization techniques and future research directions in this regard.

## Figures and Tables

**Table 1:** Communication band usage scenarios.

Communication Band	Fractional Bandwidth $B_{f}$	Band Ratio $B_{r}(f_{H}/f_{L})$
Narrow-band	$0.00<B_{f}\leq 0.01$	$0.00<B_{r}\leq 1.01$
Wide-band	$0.01<B_{f}\leq 0.25$	$1.01<B_{r}\leq 1.29$
UWB	$0.25<B_{f}<2.00$	$B_{r}\geq 1.29$

**Table 2:** UWB PHY channel definitions.

Group Band	Channel Number	Center Frequency (MHz)	Bandwidth (MHz)	Mandatory /Optional
Sub-GHz	0	499.2	499.2	Mandatory
Low	1	3494.4	499.2	Optional
	2	3993.6	499.2	Optional
	3	4492.8	499.2	Mandatory
	4	3993.6	1331.2	Optional
High	5	6486.6	499.2	Optional
	6	6988.8	499.2	Optional
	7	6489.6	1081.6	Optional
	8	7488.0	499.2	Optional
	9	7987.2	499.2	Mandatory
	10	8486.4	499.2	Optional
	11	7987.2	1331.2	Optional
	12	8985.6	499.2	Optional
	13	9484.8	499.2	Optional
	14	9984.0	499.2	Optional
	15	9484.8	1354.97	Optional

**Table 3:** Advantages and disadvantages of positioning algorithms.

Algorithm	Advantages	Disadvantages
ToA	Easy to implement. Higher scalability.	High cost. Requires precise clock.
TW-ToA	High positioning efficiency. No precise synchronization clock is required.	High cost. Longer signal processing time.
TDoA	No synchronization for anchors is required. Fewer anchors required.	High power consumption.
AoA	Provide high accuracy with short range. Complex algorithm with longer running time. Fewer anchors required.	Complex hardware design. High power consumption.
RSSI	Cost effective and low hardware complexity. No requirement for time counting devices.	Provides low precision accuracy. Requires large data for fingerprinting training.

**Table 4:** Key positioning algorithms and their description.

Paper	Year	Positioning	Algorithm	Description
[72]	2020	1D and 2D	ToA	Improving the UWB IPS accuracy by proposing a modified leading edge detection with LS trilateration filtering.
[73]	2023	3D	ToA	Proposed convolutional neural networks (CNNs) to estimate the range and then mitigated the errors by utilizing channel impulse responses (CIRs).
[74]	2021	3D	TDoA	Proposed anchor selection theory for improving the accuracy of IPS.
[75]	2022	3D	TW-ToA	A messaging framework that optimizes the usage of resources. The results showed an improvement in error to as low as 5.4 mm when using 6 anchors.
[76]	2022	2D	AoA	A fusion positioning system based on BLE-AOA and UWB was developed. It enhances the accuracy reaching below the sub-meter level.
[77]	2020	2D and 3D	RSSI	An RSSI IPS based on neural network is designed. Positioning error is <1 m and the average positioning error is 0.4436 m.

**Table 5:** Running time and confusion matrix of state-of-the-art ML algorithms for NLoS classification.

Algorithm	Running Time	LoS CR	NLoS CR	TP	FN	FP	TN	Precision	Recall	Accuracy
k-NN	0.0491 s	97.5%	79%	975	25	21	79	97.9%	97.5%	95.8%
SVM	0.1166 s	97.4%	87%	974	26	13	87	98.7%	97.4%	96.5%
DT	0.9742 s	97.7%	86%	977	23	14	86	98.6%	97.7%	96.6%
NB	0.0385 s	97.9%	88%	979	21	12	88	98.8%	97.9%	97.0%
NN	0.0606 s	98.3%	89%	983	17	11	89	98.9%	98.3%	97.5%

## Data Availability

Not applicable.

## Abbreviations

The following abbreviations are used in this manuscript:

2-Dimensional	2D
3-Dimensional	3D
Angle of Arrival	AoA
Angle of Departure	AoD
Bluetooth Low Energy	BLE
Burst Position Modulation	BPM
Channel Impulse Response	CIR
Channel State Information	CSI
Decision Tree	DT
Effective Radiated Power	ERP
Global Navigation Satellite Systems	GNSSs
Inertial Positioning System	IPS
Internet of Things	IoT
k-Nearest Neighbor	k-NN
Machine Learning	ML
Naive Bayes	NB
Neural Network	NN
Non-Line-of-Sight	NLoS
On–Off Keying	OOK
Phase of Arrival	PoA
Physical Layer	PHY
Physical Layer Header	PHR
Power Spectral Density	PSD
Pulse Amplitude Modulation	PAM
Pulse Position Modulation	PPM
Radio Frequency Identification	RFID
Received Signal Strength Indicators	RSSIs
Signal-to-Noise Ratio	SNR
Start of Frame Delimiter	SFD
Support Vector Machine	SVM
Synchronization Header	SHR
Time Difference of Arrival	TDoA
Time-of-Arrival	ToA
Time-of-Flight	ToF
Transfer Learning	TL
Two-Way Time of Arrival	TW-ToA
Ultra-Wideband	UWB
Wireless Fidelity	Wi-Fi
Wireless Personal Area Networks	WPANs
