# Correction: Che et al. Indoor Positioning System (IPS) Using Ultra-Wide Bandwidth (UWB)—For Industrial Internet of Things (IIoT). *Sensors* 2023, *23*, 5710

**DOI:** 10.3390/s23208360

**Published:** 2023-10-10

**Authors:** Fuhu Che, Qasim Zeeshan Ahmed, Pavlos I. Lazaridis, Pradorn Sureephong, Temitope Alade

**Affiliations:** 1 Department of Computing and Engineering, University of Huddersfield, Huddersfield HD1 3DH, UK; fuhu.che@hud.ac.uk (F.C.); p.lazaridis@hud.ac.uk (P.I.L.); 2 College of Arts, Media and Technology, Chiang Mai University, Chiang Mai 50200, Thailand; pradorn.s@cmu.ac.th; 3 Department of Computer Science, School of Science and Technology, Nottingham Trent University, Nottingham NG11 8NS, UK; temitope.alade@ntu.ac.uk

## Text Correction

There was an error in the original publication [1]. The previous section was more on channel impulse response, while our problem concerned making a decision on the LoS or NLoS component.

A correction has been made to “5. Detection in UWB Positioning Algorithms”, “*5.1. Machine Learning For UWB In NLoS*”:

To improve the accuracy of UWB positioning systems, specific NLoS mitigation techniques are required for various applications. Figure 13 illustrates a block diagram of a complete UWB precise IPS, which starts by fixing the anchors for the coordinate system and locates the mobile UWB tags within the indoor environment. The collected raw data will be used by an additional processing step for NLoS detection, which is performed using an ML classification algorithm that has been pre-trained with the raw measurement data. This model is used to mitigate the NLoS effects. For NLoS classification, the ML is used to classify the LoS and NLoS by different signal features or CIR values. For NLoS error correction, the ML can be applied for predicting the error $e_{i,j}$ accurately. The actual range $r_{i,j}$ between the *i*-th anchor $\left(x_{i},y_{i},z_{i}\right)$ and the *j*-th tag $\left(x_{j},y_{j},z_{j}\right)$ is defined as:

(10) $$r_{i,j}=\sqrt{{\left(x_{i}-x_{j}\right)}^{2}+{\left(y_{i}-y_{j}\right)}^{2}+{\left(z_{i}-z_{j}\right)}^{2}}.$$

The estimated distance $d_{i,j}$ in term of the coordinate of the *i*-th anchor and the *j*-th tag is determined through calculating the signal round trip time of the ToA. The positioning of the *i*-th anchors is known and constant; therefore, the estimated distance $d_{i,j}$ in a three-dimensional scenarios is given as:

(11) $$d_{i,j}^{LoS}=r_{i,j}+\epsilon_{i,j},$$

where $\epsilon_{i,j}$ is the measurement error.

For NLoS conditions, the signal direct path is reflected or blocked due to the presence of obstacles; therefore, there will be further signal propagation delay, resulting in NLoS error $e_{i,j}$. The estimated distance $d_{i,j}$ can be calculated as:

(12) $$d_{i,j}^{NLoS}=r_{i,j}+\epsilon_{i,j}+e_{i,j},$$

where $e_{i,j}$ is the independent positive measurement bias error. Finally, the corresponding ranging error $\delta_{i}$ can be expressed as:

(13) $$\delta_{i}=\left\{\begin{array}{c} \epsilon_{i,j},\;\;\;\;\;\;\;\;\;\;\;\;\;\;\;\;LoS,\\ \epsilon_{i,j}+e_{i,j},\;\;\;\;\;\;\;NLoS. \end{array}\right.$$

The ML algorithm can now be trained to classify the LoS and the NLoS component for the UWB signal. Therefore, high-accuracy NLoS classification and accurate prediction of $e_{i,j}$ is crucial for precision IPS.

With this correction, the order of some equations has been adjusted accordingly. The authors state that the scientific conclusions are unaffected. This correction was approved by the academic editor. The original publication has also been updated.
